# Self-Nanoemulsifying Drug Delivery System of Tetrandrine for Improved Bioavailability: Physicochemical Characterization and Pharmacokinetic Study

**DOI:** 10.1155/2018/6763057

**Published:** 2018-09-27

**Authors:** Chunxia Liu, Li Lv, Wei Guo, Lan Mo, Yaoxing Huang, Guocheng Li, Xingzhen Huang

**Affiliations:** ^1^Department of Pharmacy, Zengcheng District People's Hospital of Guangzhou, Guangzhou 511300, Guangdong, China; ^2^Department of Pharmacy, Sun Yat-Sen Memorial Hospital, Sun Yat-Sen University, Guangzhou 510120, Guangdong, China; ^3^School of Pharmacy, Guangxi Medical University, Nanning 530021, Guangxi, China

## Abstract

The main purpose of this study was to investigate the potential of self-nanoemulsified drug delivery system (SNEDDS) to improve the oral bioavailability of tetrandrine (Tet). SNEDDS was developed by using rational blends of excipients with good solubilizing ability for Tet which was selected based on solubility studies. Further ternary phase diagram was constructed to determine the self-emulsifying region. The optimal formulation with the best self-nanoemulsified and solubilization ability consisted of 40% (w/w) oleic acid as oil, 15% (w/w) SPC and 30% (w/w) Cremophor RH-40 as surfactant, and 15% (w/w) PEG_400_ as cosurfactant. The average droplet size and zeta-potential of the optimal Tet SNEDDS were 19.75±0.37 nm and 1.87±0.26 mv, respectively. The dissolute rate of Tet SNEDDS in various dissolution media was remarkably faster than Tet commercial tablet. Moreover, in vivo pharmacokinetic study results show that significant increase (p≤ 0.05) in the peak concentration (Cmax) and the area under the curve (AUC) of Tet was observed after the oral administration of Tet SNEDDS and the absorption of Tet from SNEDDS resulted in approximately 2.33-fold increase in oral bioavailability compared with the commercial tablet. Our research suggests that the prepared Tet SNEDDS could be a good candidate for improved the dissolution and oral bioavailability of Tet.

## 1. Introduction

**Figure 1:**
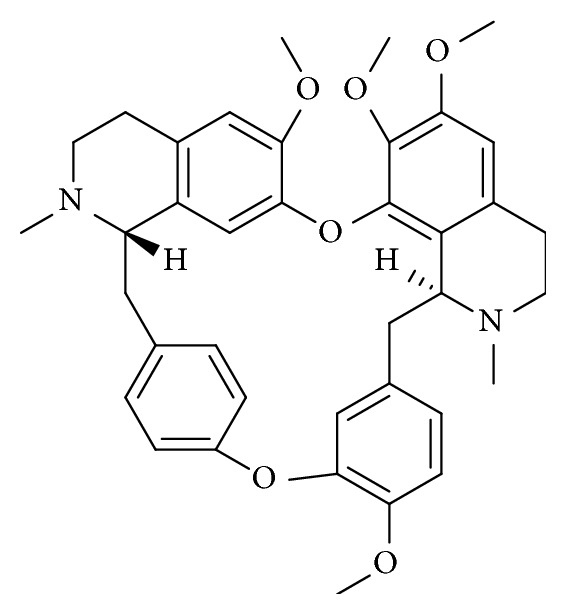
Molecular structure of tetrandrine (Tet).

Tetrandrine (Tet) is a bisbenzyltetrahydroisoquinoline alkaloid (Figure 1), which is found in the Chinese medicinal herb, han-fang-chi (or fen-fang-qi,* Stephania tetrandra S.Moore*) [1]. The first pharmacological and toxicological study of Tet was published in 1937 and demonstrated its hypotensive and cardiodepressant effects [2]. According to the literature, for over half a century, Tet has attracted large amount of interest because it shows a variety of pharmacological effects, including antiarrhythmia, antiatherogenic, antisilicosis, antihypertension, and immunomodulation effects [3–8]. Tet has been mainly used for early mild hypertension in clinical therapeutics and for severe hypertensive and hypertensive crises. The antihypertensive effect is made through a calcium antagonist mechanism [9, 10]. In recent years, Tet has also attracted much attention due to its obvious effect on experimental silicosis [11–15]. There is a growing body of evidence that Tet exerts anticancer effects against a variety of cancers, including leukemia, hepatocellular carcinoma, gastric cancer, colon cancer, lung cancer, glioma, nasopharyngeal carcinoma, prostate cancer, breast cancer, and bladder cancer [16–21].

Although Tet has potentially considerable value in clinic, some problems have arisen that have limited its clinical application. In general, a large dose (6 to 15 tablets per day) is required for the current commercially available tablets [22]. Additionally, Tet's poor solubility in physiological environment (Tet saturation=0.015 mg/mL in pH 7.4 phosphate buffered saline (PBS)) caused by the presence of a quaternary ammonium cation [23] contributes to its low and variable oral bioavailability, gastric intestine and kidney damage, and bad patient compliance [24]. In recent years, many pharmaceutical methods have been investigated to improve the bioavailability of Tet, such as lipid nanocapsules, nanoparticles, ethosomes, and microspheres [25]. However, most of tetrandrine based nanoparticles were used for injection and seldom used in oral administration.

Investigation of novel drug delivery systems to enhance the solubility and improve bioavailability of Tet is considered a matter of urgency [26]. In recent years, various strategies have been considered to overcome the problems associated with oral absorption and bioavailability such as microsphere, microcapsule, nanoemulsified drug delivery system (SNEDDS), and nanoparticles [23]. A high speed disperser and a high pressure emulsifier, generating particles with an average diameter of about 200 nm in vitro has shown some success for Tet encapsulation [27] and coencapsulated doxorubicin and bromotetrandrine lipid nanoemulsions have shown some potential of treatment in breast cancer animal models [28].

SNEDDS is an isotropic mixture of oils, surfactant, hydrophilic cosurfactant, and drug substances, which form a fine oil-in-water microemulsion when introduced into aqueous media while being gently agitated by the digestive motility of the stomach and intestine. SNEDDS is a relatively recent term used to indicate formulations with a globule size less than 100 nm [29]. It has excellent efficiency in increasing the dissolution rate, promoting oral absorption and increasing the bioavailability of poorly water-soluble drugs [30, 31], and has shown a lot of success for herbal drugs [32]. There are some commercially successful formulations such as Neoral® (cyclosporine A), Fortovase® (saquinavir), Agenerase® (amprenavir), and Norvir® (ritonavir) in clinical use.

Extensive review of literature reveals lack of reports about the bioavailability improvement of poorly water-soluble Tet using SNEDDS. Thus, the objective of the present study was to formulate Tet SNEDDS to improve Tet's oral bioavailability. To achieve this, Tet's solubility in various excipients was evaluated and that with highest solubility of Tet were selected oil, surfactant, and cosurfactant of Tet SNEDDS. The prepared Tet SNEDDS was evaluated for morphological, droplet size, zeta-potential, thermodynamic stability, and in vitro dissolution study. Furthermore, an in vivo pharmacokinetic study was performed to study Tet's pharmacokinetic behavior and bioavailability enhancement after oral administration.

## 2. Materials and Methods

### 2.1. Materials

Tet (purity>98.0%) was obtained as a gift sample from Beihai Sunshine Pharmaceutical co., Ltd. (Beihai, China). A Tet tablet (containing 20 mg Tet in one tablet with excipients including starch, dextrin, and magnesium stearate) was manufactured by Beihai Sunshine Pharmaceutical co., Ltd (Beihai, China). Oleic acid, Tween 60, and Tween 80 were purchased from Tianjin Damao Chemical Reagent Factory (Tianjin, China). Soybean oil, isopropyl acetate, olive oil, ethyl linoleate, cremophor RH-40, and isopropyl myristate (IPM) were purchased from Chengdu Gracia Chemical Technology co., Ltd (Chengdu, China). Ethyl oleate, butyl oleate, and soybean phosphatidylcholine (SPC) were purchased from Tianjin Xing Fu Fine Chemical Industry Research Institute (Tianjin, China). Isopropanol, glycerol, absolute alcohol, and PEG_400_ were purchased from Chengdu Kelon Chemical Reagent Factory (Chengdu, China). Methanol and acetonitrile (chromatographic grade) were from Thermo Fisher Scientific (China) Co., Ltd. All other chemicals used were of analytic grade.

### 2.2. Determination of Tet Solubility

The solubility of Tet was determined in selected oils, surfactants, and cosurfactants by pouring an excess of Tet into 1 mL of each excipient. The obtained mixtures were mixed using a vortex mixer. And then the mixture was kept in a water bath at 37±0.5°C for 72 hours. After equilibrium was achieved, the equilibrated samples were centrifuged at 3000 rpm for 15 min and excess insoluble Tet was discarded by filtration using a 0.45 *μ*m membrane filter. The concentration of Tet was determined by a high performance liquid chromatography (HPLC) method described below. The experiments were performed in triplicate.

### 2.3. HPLC Analysis

The amount of Tet in various excipients was quantified by a validated HPLC method [33], equipped with an ultraviolet detector (Agilent 1260 Liquid Chromatography, American). Tet was separated on a RP-C_18_ column (ODS C_18_, 200 mm×4.6 mm, 5 *μ*m) using methanol-acetonitrile–water (3 : 1 : 1, v : v : v) plus 0.06% diethylamine as mobile phase, at a flow rate of 1.0 mL/min with the detection wavelength at 282 nm. The column temperature was maintained at 25°C.

### 2.4. Preparation of SNEDDS

For preparation of Tet SNEDDS, the content of Tet was fixed at a constant level (1.0% w/w of excipients). Firstly, Tet was dissolved by cosurfactant, and then the mixture containing the calculated amount of oil and surfactant was mixed by gentle stirring for 10 min and vortex mixing until the microemulsion formulation was obtained.

### 2.5. Pseudo-Ternary Phase Diagram Study

The pseudo-ternary phase diagrams were constructed with oil, surfactant, and cosurfactant using the water titration method at room temperature: each of them representing an apex of the triangle and the total of them was kept at 100%. The mixture oil and surfactant/cosurfactant at certain weight ratio were titrated with water and mixed by magnetic stirring until equilibrium was reached. The mixtures which were clear or slightly bluish appearance were determined as microemulsions. In the SNEDDS, oleic acid was used as the oil, a mixture of SPC and cremophor RH-40 was used as surfactant and PEG_400_ was used as cosurfactant. The ratios of SPC to cremophor RH-40 by weights 1 : 1, 2 : 1, and 1 : 2 were studied by constructing a ternary phase diagram. The key factor for SNEDDS is the ratio of surfactant to cosurfactant. Phase diagrams at specific ratios of surfactant to cosurfactant by weights 1 : 1, 3 : 2, 2 : 1, and 3 : 1 were taken. Mixtures of surfactant/cosurfactant (at a specific ratio) with oil were prepared at ratios 9 : 1, 8 : 2, 7 : 3, 6 : 4, 5 : 5, 4 : 6, 3 : 7, 2 : 8, and 1 : 9 by weight. Each mixture was titrated with water and visually observed for phase clarity. The microemulsion regions in the diagrams were plotted. Also the microemulsion formulations were selected at desired components ratios.

### 2.6. Characterization of SNEDDS

#### 2.6.1. Morphological Characterization

The morphology of SNEDDS was observed by transmission electron microscopy (Hitachi transmission electron microscope H7650, Japan). SNEDDS was diluted with distilled water and mixed by slightly shaking. One drop of diluted samples was deposited on a film-coated 200 mesh copper specimen grid and allowed to stand for 20 min, after which any excess fluid was removed with the filter paper. The grid was later stained with one drop of 2% phosphotungstic acid and dried for 15 min before examination with the transmission electron microscope.

#### 2.6.2. Determination of Droplet Size and Zeta-Potential

1 mL SNEDDS was diluted with 50 mL distilled water in a glass flask and gently stirred. The droplet size distribution and zeta-potential of the resultant microemulsions were determined by dynamic light scattering with particle size apparatus (Malvern Zetasizer Nano ZS90, UK). Each sample was analyzed in triplicate.

#### 2.6.3. Thermodynamic Stability Study

The objective of thermodynamic stability is to evaluate the phase separation and effect of temperature variation on SNEDDS formulation. The stability was evaluated by centrifugation, a heating-cooling cycle and a freeze-thaw cycle. Briefly, Tet was diluted with aqueous medium, followed by centrifugation at 15000 rpm for 15 min, and then the mixture was observed visually for phase separation; after SNEDDS formulations were diluted with deionized water (1 : 50) and subjected to freeze-thaw cycles (-20°C for 2 days) followed by heated to 40°C for 2 days, the phase separation and appearance were observed.

#### 2.6.4. In Vitro Dissolution Study

The in vitro dissolution behaviors of Tet from commercial tablet of Tet and Tet SNEDDS were assessed using the Chinese pharmacopoeia (2010 version) paddle method. The SNEDDS containing Tet (1.0% w/w of the excipients) were added into hydroxy-propyl methyl cellulose (HPMC) capsules “size 0.” A Tet SNEDDS hard capsule/tablet was put into a sinker. Thus, the sinker was loaded with 900 mL of simulated gastric fluid without enzymes (pH 1.2), phosphate buffer pH 6.8, and distilled water (containing 0.5% of sodium dodecyl sulfate to increase dissolution) at 37±0.5°C with a paddle speed of 100 rpm using a dissolution tester (ZRS-8G, Tianjin University Electronics Co. Ltd, Tianjin, China). 5.0 mL sample was withdrawn at 5, 10, 20, 30, 45, 60, 90, 120, and 180 min and replaced with fresh dissolution medium to keep the volume constant. The release of Tet from the SNEDDS formulation was compared with commercial tablets containing the same quantity of drug. The concentration of Tet in the released sample was determined by the HPLC method described in the above section “2.3 HPLC analysis.”

### 2.7. Pharmacokinetic Study

The pharmacokinetic experiment was designed to compare the Tet SNEDDS formulation and the Tet commercial tablet. The male Wistar Albino rats (180-220 g) were purchased from Guangxi Medical University Laboratory Animal Centre, China. They were allocated to two groups at random: the first group received the Tet commercial tablet, which was prepared by Tet suspension (0.25% w/v carboxymethyl cellulose sodium (CMC-Na)) according to the following method: ten tablets were ground to a fine powder with a mortar. The powder was weighed and mixed with 0.25% CMC-Na solution to make a suspension. The suspension was made and used when needed. The second group received the Tet SNEDDS formulation. Each group consisted of six rats. Tet SNEDDS formulation was given orally or gavage at the same dose of 10 mg/kg body weight. The dose was based on the median of the clinical dose of Tet tablets, which is between 80 and 120 mg/day/person. Based on an average weight of 60 kg per person, the dose will be 1.67 mg/kg/day. Thus, for the rat model the dose was 10 mg/kg/day. All rats were dosed following an overnight fast. Food was supplied to the rats after 4 h of the dose administration. In vivo study was approved by the Animal Ethics Committee of Guangxi Medical University.

Blood samples (approximately 0.3 mL) were collected from the retroorbital plexus with a heparinized tube at predose and 0.25, 0.5, 1, 2, 3, 4, 6, 8, 12, 24, 48, and 72 h postdose. Blood samples were centrifuged at 6000 rpm for 10 min. 5 *μ*L demethyltetrandrine (internal standard, IS) solution (10 *μ*/mL), 0.1 mL acetonitrile and 0.4 mL ether were added to 0.1 mL upper plasma. After vortex mixing for 3 min and centrifugation at 3000 rpm for 15 min, the upper organic layer was transferred and the plasma was extracted by 0.4 mL ether again. The combined organic phase was evaporated to dryness at 40°C. Then the residue was dissolved in 100 *μ*L methanol and centrifuged at 10000 rmp for 10 min [34]. After centrifugation, 2 *μ*L of clear supernatant was injected into UPLC-MS-MS system for analysis.

The concentration of Tet was determined by UPLC-MS-MS according to the following: HPLC analysis was performed using the Dionex UltiMate 3000 with a binary pump, an on-line degasser, an auto-sampler, and a column temperature controller. Chromatographic separations were performed on a Hypersil GOLD C_18_ column (100 mm × 2.1 mm, 1.9 *μ*m) protected by a C_18_ guard column (10 mm × 2.1 mm, 5 *μ*m) at 35°C. The mobile phase consisted of methanol–water (50 : 50, v/v). The flow rate was set at 0.2 mL/min.

MS analysis was carried out on a Thermo Scientific TSQ Quantum Access MAX triple stage quadrupole mass spectrometer with an electrospray ionization (ESI) source running in a positive-ionization mode. The typical ion source parameters were spray voltage: 3500 V; sheath gas pressure (N_2_): 30 units; auxiliary gas pressure (N_2_): 10 units; ion transfer tube temperature: 400°C; collision gas (Ar): 1.5 mTorr; Q1/Q3 peak resolution: 0.7 Da; scan width: 0.002 Da; samples were analyzed via selective-reaction monitoring (SRM) with monitoring ion pairs at m/z 623 → 381 for Tet, m/z 609 → 367 for IS. The scan dwell time was set at 0.1s for every channel. All data collected in centroid mode were acquired and processed using Xcalibur 2.2 software (Thermo Fisher Scientific Inc., USA). Peak area ratio of Tet to IS was calculated, and the calibration curve was established with the ration as Y-axis while the corresponding nominal concentrations of Tet as X-axis.

### 2.8. Data Analysis

Statistical evaluation was performed by Student t-test of paired observations to analyze different concentrations of Tet. The pharmacokinetic data were processed by noncompartmental analysis using the DAS 2.0 software package (Chinese Pharmacological Society).

## 3. Results

### 3.1. Solubility Studies of Tet in Various Excipients

The objective of solubility studies is to identify the most suitable oil, surfactant, and cosurfactant which have a good solubilizing capacity for Tet. The concentration of Tet in various excipients at 37°C was determined by HPLC, and solubility results are presented in Table 1. If excipients with high drug solubility are chosen to prepare SNEDDS formulation, then the amount of formulation to be administered will be small. It is clear from the table that the highest solubility of Tet is in oleic acid as the oil and in the surfactants, SPC, and cremophor RH-40, with the cosurfactant, PEG_400_, and absolute alcohol. However, absolute alcohol was not selected as cosurfactant due to its volatility that would result in precipitation or crystallization of drugs. So in the preparation of SNEDDS, oleic acid was used as oil, SPC and cremophor RH-40 were used as surfactant, and PEG_400_ was used as cosurfactant.

### 3.2. Construction of Pseudo-Ternary Phase Diagrams

**Figure 2:**
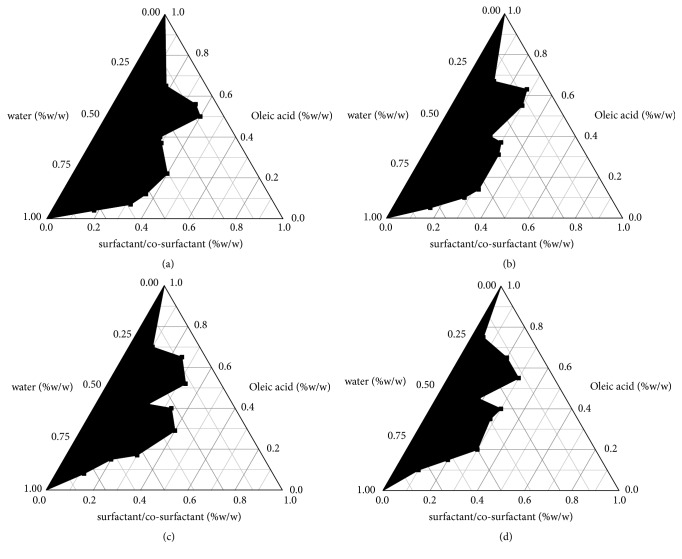
Pseudo-ternary phase diagram consisting of oleic acid, the mixture of surfactant (SPC/cremophor RH-40, 1 : 2), and PEG_400_ with surfactant/cosurfactant ratios of (a) 1 : 1, (b) 3 : 2, (c) 2 : 1, and (d) 3 : 1. The blank region represents oil/water microemulsion formation area.

Pseudo-ternary phase diagrams were constructed to identify the microemulsion regions for optimizing the concentrations of oil, surfactant, cosurfactant, and their mixing ratios. The visual test measured the apparent spontaneous of emulsion formation. Screening of the mixed surfactant was based on the pseudo-ternary phase diagram. As shown in Table 1, for surfactant, Tet had the highest solubility in cremophor RH-40, followed by SPC. The maximum region of self-microemulsion was obtained when mixture of SPC and cremophor RH-40 in the ratio of 1 : 2 was used. Therefore, the optimal of SPC to cremophor RH-40 was selected to be 1 : 2. The phase diagrams containing oleic acid, SPC and cremophor RH-40, and PEG_400_ were shown in Figure 2. As shown in Figure 2, microemulsion formation area was increased with an increase in 3 : 1 (the ratio of surfactant to cosurfactant) and 3 : 2 (the ratio of mixtures including surfactant and cosurfactant to oil). There will be a loss of flow if the surfactant to cosurfactant ratio is more than 3 : 1. According to these results, the following SNEDDS formulation was established: 40% (w/w) oleic acid as oil, 15% (w/w) SPC and 30% (w/w) Cremophor RH-40 as surfactant, and 15% (w/w) PEG_400_ as cosurfactant.

### 3.3. Characterization of SNEDDS

#### 3.3.1. Morphological Characterization

**Figure 3:**
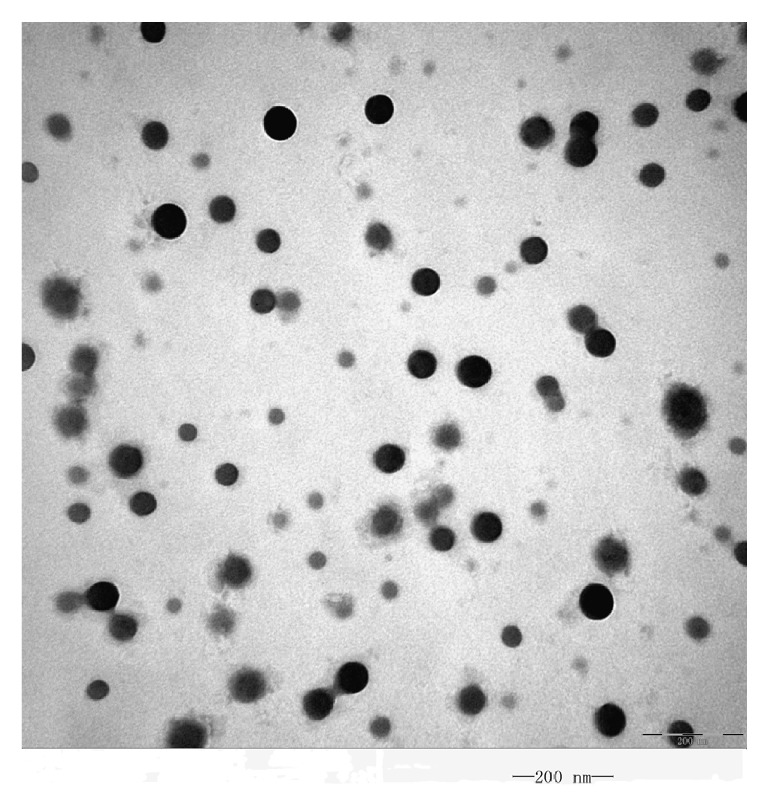
Transmission electron microscopy photo of Tet microemulsion (×100000).

In order to observe the oily droplets, the SNEDDS of Tet was turned into microemulsion by diluting with distilled water. A representative transmission electron microscope picture is shown in Figure 3. From the figure it is evident that all the oil droplets were of spherical in shape.

#### 3.3.2. Droplet Size and Zeta-Potential Analysis

**Figure 4:**
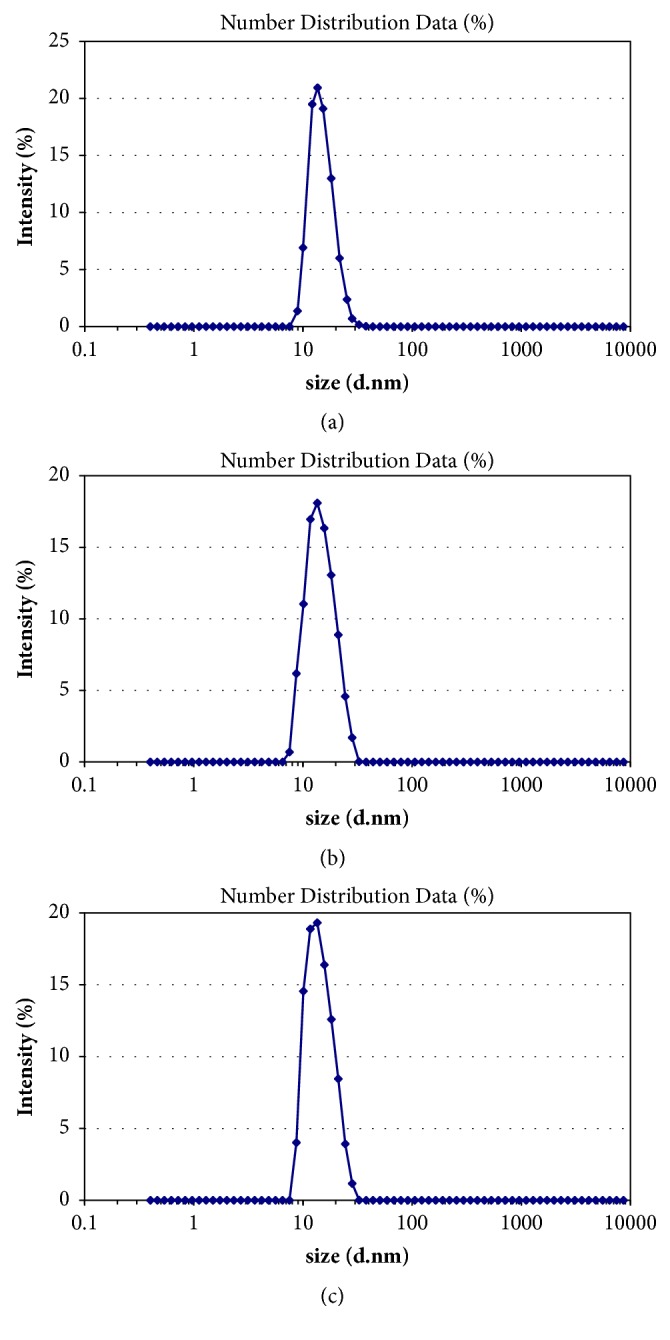
(a–c) Droplet size distribution by intensity (n=3).

**Figure 5:**
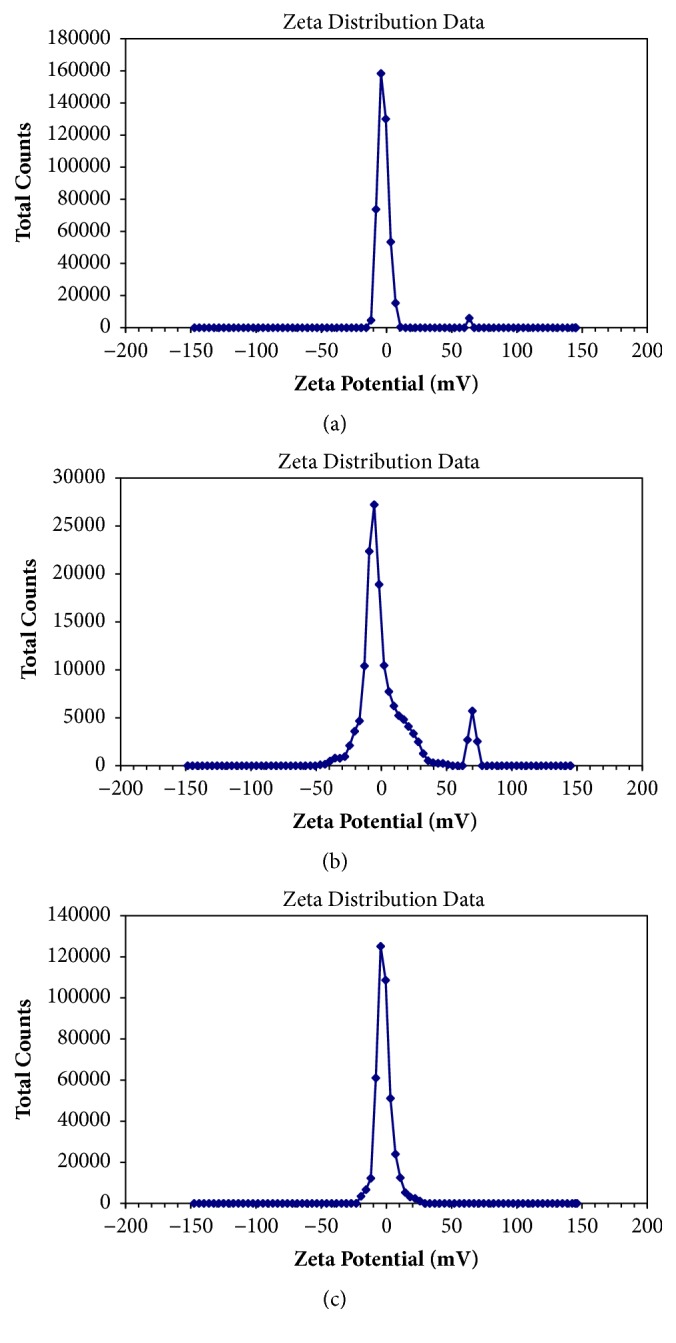
(a–c) zeta- potential distribution (n=3).

As shown in Figure 4, the mean droplet sizes of diluted SNEDDS preconcentrates were very low, and all were found to be in the nanometric range (<100 nm). The average droplet size of Tet microemulsion was 19.75±0.37 nm and showed Gaussian distribution. The SNEDDS of Tet was diluted with distilled water. The resultant zeta-potential was 1.87±0.26 mv, which indicated that the microemulsion droplets had no charge (see Figure 5).

#### 3.3.3. Thermodynamic Stability Studies

For lipid-based dosage forms, stability is crucial to their performances which can make an adverse effect in the form of precipitation of the drug in the excipient matrix. In thermodynamic stability studies, formulation selected was subjected to stress test like centrifugation (at 15000 rpm for 15 min) and freeze-thaw test (-20°C for 2 days followed by +40°C for 2 days). The SNEDDS formulation was found to be stable under these stressed conditions. Since the Tet SNEDDS formulation was stable, a metastable formation is thus avoided and frequent tests need not be performed during storage.

#### 3.3.4. In Vitro Dissolution Study

**Figure 6:**
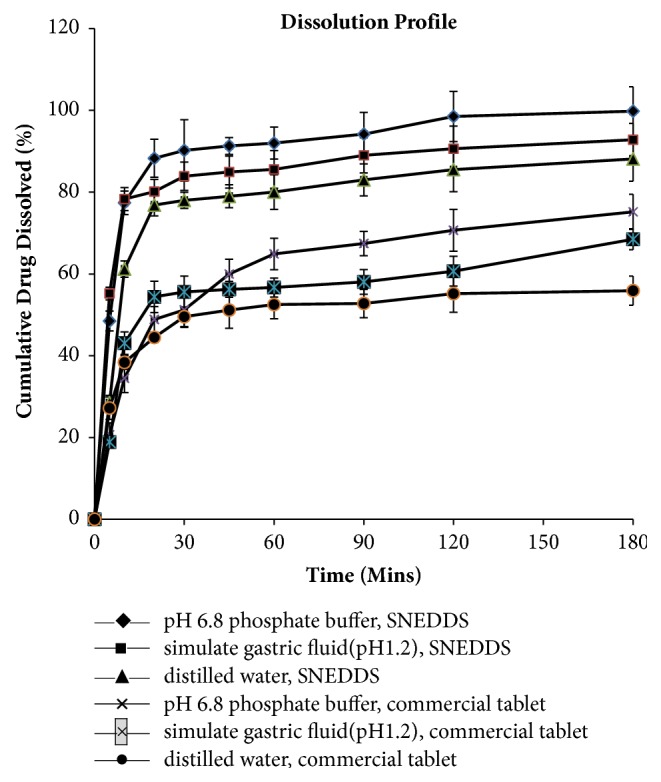
In vitro profiles of Tet from SNEDDS and Tet commercial tablet formulation in various medium at 37°C with a paddle speed of 100 rpm using a dissolution tester (ZRS-8G, Tianjin University Electronics Co. Ltd, Tianjin, China). Each value represents the mean ± SD (n=6).

An in vitro dissolution profile of the optimized Tet SNEDDS formulation was performed to compare with the commercial tablet, and the dissolution of drug from various media was evaluated. The dissolution of Tet from SNEDDS and Tet commercial tablet formulation in various medium were presented in Figure 6 and showed that the Tet SNEDDS formulation allowed 85% dissolution of Tet within 20 min whereas the commercial tablet showed less than 80% of Tet within 180 min. The dissolute rate of the Tet SNEDDS formulation in various dissolution media was remarkably faster than commercial tablet due to the small droplet size. In order to establish the kinetics of drug dissolution, various mathematical models including zero-order, first-order, Higuchi, Korsmyer-Peppas, Hixson-Crowell, and Weibull were used. The assessment of the models is presented in Table 2 that provides the model selection criteria (MSC), by selecting the model with the highest MSC, it was found that the drug dissolution from SNEDDS best fitted with Weibull Distribution Mode: *y* = 5.67 × 10^6^ × (1 − *e*(−(*x* − 4.99)^0.05^/(7.99 × 10^4^))), r^2^ = 0.9996.

### 3.4. Pharmacokinetic Study

**Figure 7:**
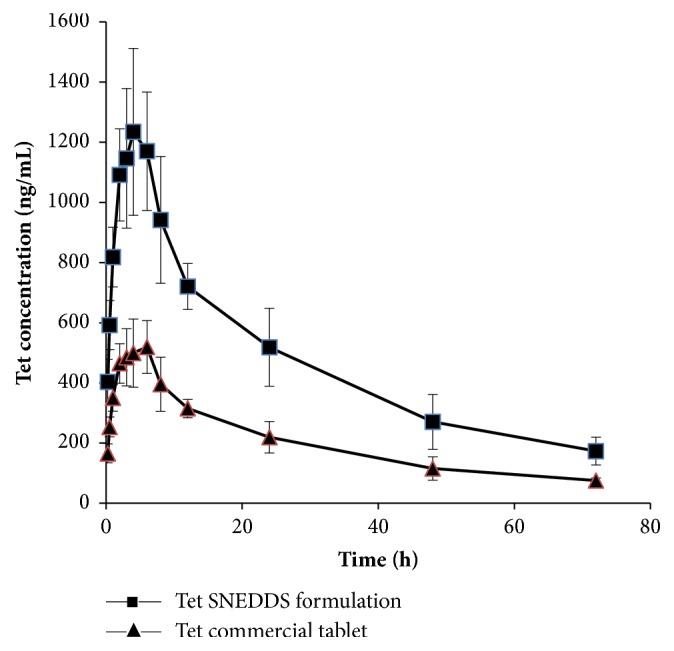
Plasma concentration profile of Tet after oral administration in male rats. Each value represents the mean ±S D (n=6 and 10 mg/kg).

Figure 7 presents the plasma concentration vs time curve for Tet SNEDDS formulation and Tet commercial tablet after a single dosage of oral administration in male rats. At all the indicated time points, Tet blood concentrations in rats treated with Tet SNEDDS were significantly higher than those treated with Tet commercial tablet. Pharmacokinetic parameters are shown in Table 3. The results indicated that the average of T_max_ of the SNEDDS formulation and commercial tablet was 3.8 h and 6.6 h, respectively, which meant that the Tet SNEDDS formulation showed a faster rate of absorption than the Tet commercial tablet. The peak plasma concentration (C_max_) and the area under the curve (AUC_0-72 h_) of SNEDDS of Tet were higher than the Tet commercial tablet by 137.8% and 134.1%, respectively. The relative bioavailability of the Tet SNEDDS formulation was 2.33-fold compared with the Tet commercial tablet. This indicated better systemic absorption of Tet from the SNEDDS formulation compared to the commercial tablet. The Tet SNEDDS formulation demonstrated almost similar mean residence time (MRT) (39.6 h) compared to the Tet commercial tablet MRT (39.9 h). Therefore, no prolonged action was found in the SNEDDS formulation. It was reasonable to conclude that SNEDDS enhances the absorption in vivo significantly.

## 4. Discussion

In our research, the potency of a self-microemusifying drug delivery system was successfully investigated in order to provide an effective system for delivery of Tet. Based on the results of the solubility tests in various excipients, we selected the optimal oil, cosolvent, surfactant, and cosurfactant. Through the construction of a ternary phase diagram, the optimal formulation of SNEDDS containing Tet was found to be the following: 40% (w/w) oleic acid as oil, 15% (w/w) SPC and 30% (w/w) Cremophor RH-40 as surfactant, and 15% (w/w) PEG400 as cosurfactant. The particle size, zeta-potential, stability, in vitro dissolution, and in vivo bioavailability of the resultant emulsion after self-emulsification were determined. The average droplet size of the optimal formulation was 19.75±0.37 nm, and the average zeta-potential was 1.87±0.26 mv. With other new formulations of Tet such as nanoparticles (Tet-loaded PVP-b-PCL nanoparticles) [35], nanocapsules (tetrandrine-phospholipid complex loaded lipid nanocapsules) [22], and microsphere (tetrandrine-loaded chitosan microsphere) [25], SNEDDS-Tet has a smaller particle size (size ranges of nanocapsules and microsphere: from 40 nm to 15 *μ*m), equivalent solubility (solubility of nanocapsules in water: 8.28 ± 0.37 *μ*g/mL), and a longer mean residence time (MRT of nanocapsules nanocapsules: 8.39 ± 1.532 h).

Encapsulation of drugs that are poorly soluble and have hydrophobic properties and poor distribution has been studied quite widely [31, 36]. This study has some advantages over previous studies into the delivery of Tet because the SNEDDS-Tet can form a microemulsion spontaneously and the diameter of SNEDDS-Tet was smaller than those used previously [27], which might allow it to be more easily delivered into cells or to pass across some barriers. Recently, in order to overcome high cost of SNEDDS, a new, supersaturable self-emulsifying drug delivery system (S-SEDDS) was developed [37]. But some preparation methods of S-SEDDS cannot be used for Tet because of its thermal instability. Therefore, the focus of our research for Tet concentrates on SNEDDS.

Using mixed surfactants can achieve a rational value of hydrophile-lipophile balance (HLB) that using a single surfactant cannot achieve. The mixture of cremophor RH-40 and SPC can adjust the arrangement of the surfactant in the oil-water interface and improve the structure of the interfacial film, increase mobility and stability of the film, and so allow easier formation of a microemulsion [38]. In addition, selecting the appropriate surfactant HLB value can reduce the appearance of crystallization. The expected HLB of SNEDDS is usually within the range of 14-18, and the HLB of SNEDDS was 14-16 in this study. In vitro dissolution experiments revealed that the dissolution of Tet from SNEDDS was faster than the commercial tablet. The relative bioavailability of Tet SNEDDS was enhanced by 233%. In addition to improving the solubility of the drug, there are a number of other biopharmaceutical properties that can affect the bioavailability of hydrophobic drugs. Surface active agents, dispersed particle size, lymphatic transport, lipolysis, and inhibition of intestinal metabolism can improve bioavailability. Thus, the system developed here could be used as an effective approach for enhancing the solubility and bioavailability of Tet and the Tet SNEDDS is worthy of further investigation.

## 5. Conclusion

In summary, self-nanoemulsifying drug delivery system composed of 40% (w/w) oleic acid as oil, 15% (w/w) SPC and 30% (w/w) Cremophor RH-40 as surfactant, and 15% (w/w) PEG_400_ as cosurfactant was used to prepare the Tet SNEDDS. The results of in vitro dissolution study show that the dissolute rate of the prepared Tet SNEDDS in various dissolution media was remarkably faster than commercial tablet due to the small droplet size. And the results of pharmacokinetic study show that the prepared Tet SNEDDS achieved an approximately 2.33-fold increase in bioavailability compared with the commercial tablet. It could be concluded that self-nanoemulsifying drug delivery system of tetrandrine has the potential to improve the dissolution and oral bioavailability of poor water-soluble Tet.

## Figures and Tables

**Table 1:** Result of solubility studies with Tet in various excipients (n=3).

Sample	Excipient	Function in SNEDDS	Solubility(mg/mL)
1	Oleic acid	Oil	8.674±0.056
2	Soybean oil	Oil	2.372±0.091
3	Isopropyl acetate	Oil	2.291±0.117
4	Olive oil	Oil	2.124±0.065
5	Ethyl linoleate	Oil	3.326±0.177
6	Isopropyl myristate (IPM)	Oil	4.348±0.198
7	Ethyl oleate	Oil	1.185±0.066
8	Butyl oleate	Oil	1.194±0.064
9	Tween-60	Surfactant	4.216±0.208
10	Tween-80	Surfactant	6.793±0.324
11	SPC	Surfactant	10.238±0.199
12	Cremophor RH-40	Surfactant	12.645±0.252
13	Isopropanol	Co-surfactant	2.564±0.088
14	Glycerol	Co-surfactant	3.455±0.100
15	PEG400	Co-surfactant	9.584±0.225
16	Absolute alcohol	Co-surfactant	8.449±0.165

**Table 2:** The model selection criteria of various mathematical models for the drug dissolution from SNEDDS.

Mathematical models	Model selection criteria (MSC)
Zero-order	-1.3324
First-order	1.6415
Higuchi	-0.2989
Korsmyer-Peppas	3.0797
Hixson-Crowell	-0.2690
Weibull	5.7438

**Table 3:** Pharmacokinetic parameters of Tet SNEDDS formulation and Tet commercial tablet after single dose administration (n=6).

Parameters	Unit	SNEDDS	Tablet
AUC_0-72 h_	ng/mL h	33855.6±2024.3^*∗*^	14459.4±3131.4
AUC_0-*∞*_	ng/mL h	41078.4±3621.2^*∗*^	17604.0±4337.3
T_max_	h	3.8±1.2^*∗*^	6.6±1.6
C_max_	ng/mL	1234.8±39.7^*∗*^	519.3±26.8
MRT	h	39.6±10.2	39.9±12.1
Relative bioavailability	233.3%		

^*∗*^ *p* <0.05 compared to the Tet commercial tablet.

## Data Availability

The data used to support the findings of this study are available from the corresponding author upon request.
